# A phase I trial of metformin in combination with vincristine, irinotecan, and temozolomide in children with relapsed or refractory solid and central nervous system tumors: A report from the national pediatric cancer foundation

**DOI:** 10.1002/cam4.5297

**Published:** 2022-09-23

**Authors:** Jonathan L. Metts, Matteo Trucco, Daniel A. Weiser, Patrick Thompson, Eric Sandler, Tiffany Smith, Jessica Crimella, Samer Sansil, Ram Thapa, Brooke L. Fridley, Nicholas Llosa, Thomas Badgett, Richard Gorlick, Damon Reed, Jonathan Gill

**Affiliations:** ^1^ Cancer and Blood Disorders Institute Johns Hopkins All Children's Hospital St Petersburg Florida USA; ^2^ Cleveland Clinic Children's Hospital Department of Pediatric Hematology‐Oncology & Bone Marrow Transplantation Cleveland Ohio USA; ^3^ Departments of Pediatrics and Genetics, Montefiore Medical Center Albert Einstein College of Medicine Bronx New York USA; ^4^ Division of Pediatric Hematology‐Oncology University of North Carolina Health Care Chapel Hill North Carolina USA; ^5^ Department of Pediatric Oncology Nemours Health Systems Jacksonville Florida USA; ^6^ Cognitive Research Corporation St Petersburg Florida USA; ^7^ Clinical Trials Office Partnerships H. Lee Moffitt Cancer Center and Research Institute Tampa Florida USA; ^8^ Cancer Pharmacokinetics and Pharmacodynamic Core H. Lee Moffitt Cancer Center and Research Institute Tampa Florida USA; ^9^ Department of Biostatistics and Bioinformatics H. Lee Moffitt Cancer Center and Research Institute Tampa Florida USA; ^10^ Department of Oncology, Sidney Kimmel Comprehensive Cancer Center Johns Hopkins University School of Medicine Baltimore Maryland USA; ^11^ Department of Pediatrics University of Kentucky College of Medicine Lexington Kentucky USA; ^12^ Department of Pediatrics University of Texas MD Anderson Cancer Center Houston Texas USA; ^13^ Adolescent and Young Adult Program, Department of Interdisciplinary Cancer Management H. Lee Moffitt Cancer Center Tampa Florida USA

**Keywords:** irinotecan, metformin, pediatric, phase I, temozolomide, vincristine

## Abstract

**Background:**

Patients with relapsed and refractory solid and central nervous system (CNS) tumors have poor outcomes and need novel therapeutic options. Vincristine, irinotecan, and temozolomide (VIT) is a common chemotherapy regimen in relapsed pediatric tumors with an established toxicity profile. Metformin shows preclinical anti‐cancer activity through multiple pathways.

**Methods:**

The objective of this Phase I trial was to establish the maximum tolerated dose (MTD) and recommended Phase II dose (RP2D) of metformin in combination with VIT in children with relapsed and refractory solid and CNS tumors. A 3 + 3 design was used to test the addition of metformin at five dose levels (666, 999, 1333, 1666, and 2000 mg/m^2^/day). Therapy toxicity, pharmacokinetics, and radiologic response to treatment were evaluated.

**Results:**

Twenty‐six patients (median age 13 years, range 2–18 years) were enrolled with 22 evaluable for toxicity. The most common diagnoses were Ewing sarcoma (*n* = 8), rhabdomyosarcoma (*n* = 3) and atypical teratoid/rhabdoid tumor (*n* = 3). The MTD was exceeded at Dose Level 5 due to two dose‐limiting toxicities; both were Grade 3 diarrhea requiring prolonged hospitalization and intravenous fluids. The MTD was not determined due to study closure with less than six patients enrolled at Dose Level 4. Frequently observed toxicities were gastrointestinal (most notably diarrhea) and hematologic. Amongst 16 patients evaluable for best overall response, there was one complete response (Ewing sarcoma), three partial responses (Ewing sarcoma, glioblastoma multiforme, and alveolar rhabdomyosarcoma), and five patients with stable disease.

**Conclusions:**

The MTD of VIT with metformin was not determined due to premature study closure. We recommend an RP2D of Dose Level 4, 1666 mg/m^2^/day. Radiographic responses were seen in multiple tumor types. Further evaluation for efficacy could be investigated in a Phase II trial.

**Abbreviations**
ALL: Acute Lymphoblastic Leukemia
AUC: Area Under Curve
CNS: Central Nervous System
Css: Concentration at Steady State
CR: Complete Response
DLT: Dose‐limiting Toxicity
FDA: Food and Drug Administration
GI: Gastrointestinal
IRB: Institutional Review Board
IV: Intravenous
MTD: Maximum Tolerated Dose
ORR: Overall Response Rate
PD: Progressive Disease
PK: Pharmacokinetic
PR: Partial Response
SD: Stable Disease
VIT: Vincristine, Irinotecan and temozolomide

## 1 BACKGROUND

Solid tumors, including central nervous system (CNS) tumors, represent approximately 60% of all childhood malignancies and account for the majority of cancer deaths in children.^1^ Modest improvements in survival have been observed in children with these tumors over the past twenty years.^2^ These disappointing results have redoubled efforts to find active agents to combine with traditional chemotherapy, including the repurposing of FDA‐approved medications.

Irinotecan and temozolomide are frequently used in pediatric solid and CNS tumors due to clinical tolerability and preclinical evidence of synergy.^3^, ^4^, ^5^, ^6^, ^7^ Vincristine, irinotecan and temozolomide (VIT) was studied in two Phase I trials in children with refractory or relapsed disease, with radiographic responses and prolonged stable disease seen in sarcomas, neuroblastoma, and CNS tumors.^8^, ^9^ A randomized Phase II trial enrolling mostly children demonstrated an improve overall survival in relapsed rhabdomyosarcoma patients receiving VIT compared to vincristine and irinotecan.^10^ Common toxicities of this regimen are hematologic toxicity and irinotecan‐induced diarrhea which can be managed through supportive care including prophylaxis with cephalosporin antibiotics. This well‐tolerated regimen provides a useful backbone to study the addition of novel agents.

Metformin is an FDA‐approved oral biguanide used to treat type II diabetes. An international randomized placebo‐controlled trial showed metformin was safe and effective in children 12 and older, with main side effects of abdominal pain and nausea/vomiting.^11^ Metformin has preclinical activity against multiple models of human cancer through multiple mechanisms. Metformin activated the AMP‐activated protein kinase (AMPK) pathway in malignant and nonmalignant tissues, resulting in growth inhibition in several models including breast and p53‐deficient colon cancer.^12^, ^13^, ^14^ In breast and prostate cancer models, metformin induced inhibition of mammalian target of rapamycin (mTOR) downstream of AMPK and through REDD1 (regulated in development and DNA damage responses 1).^15^, ^16^ Through inhibition of mitochondrial complex I, metformin in combination with glutaminase inhibition inhibited growth of primary tumors and reduced frequency of lung metastases in a murine osteosarcoma model.^17^, ^18^ Epidemiologic data suggests metformin provided patients treated for diabetes with protection against development of cancer.^19^, ^20^, ^21^, ^22^ The study of metformin for cancer therapy must take into consideration the dosing strategy of metformin in preclinical studies and its volume of distribution. Metformin concentrations above 1 mM are generally used for in vitro cancer experiments. While mouse peak plasma metformin concentrations after IV injections of metformin are much higher than oral administration, the oral route results in more consistent delivery of metformin to tumors.^23^ In adult clinical trials, plasma concentrations up to 25 μM at oral doses up to 2500 mg/day have been observed.^23^ In regards to potential therapy for solid tumors, metformin has a large volume of distribution of 63–276 liters when given IV, indicating a considerable tissue uptake, which has been confirmed in mouse models.^24^

Phase I studies of metformin alone and in combination with other agents in adults with cancer have found a wide range of tolerable metformin doses as high as 2000 mg/day. However in one study in combination with temsirolimus, the starting dose level was not tolerated due to dose limiting toxicities of pneumonitis, fatigue and thrombocytopenia, requiring a dose reduction of temsirolimus and a maximum metformin dose of only 500 mg/day.^25^, ^26^, ^27^ The Sunshine Project previously reported a Phase I study of metformin in combination with vincristine, dexamethasone, PEG‐asparaginase, and doxorubicin in relapsed and refractory pediatric acute lymphoblastic leukemia (ALL), which demonstrated a metformin maximum tolerated dose (MTD) and recommended Phase II dose (RP2D) of 1000 mg/m^2^/day.^28^ Concurrently to the ALL trial, and based on preclinical evidence of metformin anticancer activity and the lack of established dosing tolerance in adults, we sought to determine the safety and tolerability of metformin when combined with VIT in relapsed and refractory pediatric solid and CNS tumors.

## 2 MATERIALS AND METHODS

### 2.1 Objectives

The study's primary objectives were to determine the MTD and RP2D of metformin combined with VIT in pediatric relapsed and refractory solid and CNS tumors, and to describe pharmacokinetics (PKs) of metformin in combination with VIT. The secondary objective was to describe the radiologic responses seen with this regimen.

### 2.2 Patient eligibility

Patients 1–18 years of age with histologically or radiographically confirmed relapsed or refractory CNS or non‐CNS solid tumors with radiographically measurable disease and no known curative therapy options were eligible. A Karnofsky/Lansky score of 50 or above was required. Prior therapy including vincristine, irinotecan, or temozolomide was permitted, however patients could not have previously received irinotecan and temozolomide in combination. Prior radiation therapy was allowed if completed greater than 14 days prior to the start of protocol therapy for local palliative radiation and greater than six months for total body or craniospinal irradiation. Patients with prior autologous or allogenic stem cell transplant were eligible if more than three months from engraftment, transfusion independent, without graft versus host disease and not taking immunosuppressive medications. Organ function requirements included an absolute neutrophil count ≥1000/mm^3^, platelet count ≥100,000/mm^3^ (with no platelet transfusion within seven days of eligibility labs), hemoglobin ≥8.0 gm/dl, calculated or measured creatinine clearance or glomerular filtration rate ≥70ml/min/1.73 m^2^, total bilirubin ≤1.5 × upper limit of normal for age, alanine aminotransferase ≤5 × upper limit of normal for age, and serum albumin ≥2 gm/dl.

Study exclusions included pregnant or breast‐feeding women, prior allergy or intolerance of vincristine, irinotecan, temozolomide, or metformin, allergy to cephalosporins, or uncontrolled infection. Other exclusions included ongoing treatment with other investigational agents, other concomitant anti‐cancer agents, or hematologic growth factors. CNS tumor subjects on dexamethasone were required to be on a stable or decreasing dose for minimum seven days before enrollment.

### 2.3 Study design

This study was completed through the Sunshine Project, a multi‐institutional clinical trial consortium sponsored by the National Pediatric Cancer Foundation. Moffitt Cancer Center was the coordinating center, and the study was approved by the Moffitt Cancer Center Institutional Review Board (IRB) and each participating institution's IRB. The trial was registered at www.clinicaltrials.gov (NCT01528046). Written informed consent and assent was obtained according to institutional guidelines.

Dose escalation followed a 3 + 3 design.^29^ Common Terminology Criteria for Adverse Events (CTCAE) version 4.0 was used for toxicity grading. All Grade 3 and 4 toxicities and any toxicities possibly, probably, or definitely attributed to metformin were collected. Dose‐limiting toxicity (DLT) evaluation occurred during the first metformin‐containing cycle of treatment, and patients were required to complete a minimum 80% of prescribed metformin doses or to experience a DLT at any time in the cycle to be evaluable for DLT. DLT was defined as any Grade 3 or 4 non‐hematologic toxicity possibly, probably, or definitely attributable to the investigational drug with exclusion of the following Grade 3 events: allergic reactions, nausea, vomiting, dehydration, diarrhea requiring intravenous hydration for 48 h or less, diarrhea not requiring intravenous fluids lasting five days or less, aspartate aminotransferase or alanine aminotransferase elevation returning to Grade ≤1 or baseline prior to the next treatment course, fever, febrile neutropenia, infection, electrolyte abnormalities improving to ≤ Grade 2 within seven days with or without supplements, alopecia, or vincristine‐related neuropathy. The following hematologic toxicities were considered DLTs: Grade 4 neutropenia more than 14 days duration, Grade 3 or 4 thrombocytopenia more than 14 days duration, and failure to recover blood counts to eligibility criteria causing a delay more than 21 days between treatment courses. The MTD of metformin was exceeded if two or more patients in a cohort up to six patients at a given dose level experienced DLT. Once determined, we planned to declare the MTD as the RP2D.

Patients were evaluated with cross‐sectional imaging within 14 days of therapy initiation and before course three, five, and every three cycles thereafter. Responses were evaluated using the Response Criteria in Solid Tumors.^30^ To be evaluable for response, a minimum 21 days of metformin therapy with a minimum 80% of metformin doses taken or documented progressive disease after metformin initiation was required. An overall best response assessment required two consecutive determinations of disease status separated by a minimum of three weeks. Responses were characterized as complete response (CR), Partial response (PR), Stable Disease (SD), and Progressive Disease (PD).

### 2.4 Treatment schema

**FIGURE 1:**
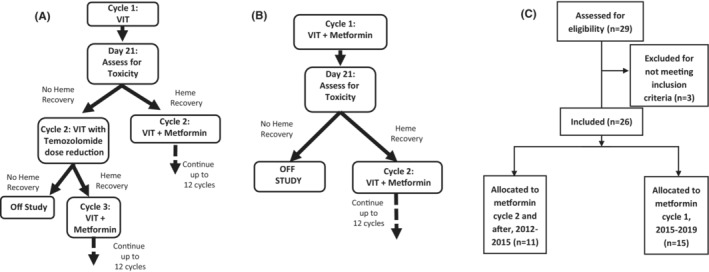
Trial design: (A) Schema for the original trial VIT cycle without metformin prior to addition of metformin in Cycle 2 (or 3) depending on hematologic tolerance. “Heme Recovery” signifies ANC > 1000 and Platelets >100,000 on Day 21 of a cycle. (B) Post‐amendment schema showing the addition of metformin in Cycle 1. (C) Trial profile accounting for all patients screened for enrollment.

Initially, protocol therapy included VIT (vincristine 1.5 mg/m^2^ intravenous Days 1 and 8, irinotecan 50 mg/m^2^ intravenous Days 1–5, temozolomide 100 mg/m^2^ oral Days 1–5 in a 21‐day cycle) alone in Cycle 1, with metformin given concurrently beginning in Cycle 2 if the patient had adequate hematologic recovery (absolute neutrophil count ≥1000/mm^3^ and platelets ≥100,000/mm^3^) on Day 21 of Cycle 1. If this was not achieved, then a second cycle of VIT was given with a temozolomide dose reduction to 50 mg/m^2^/dose. If adequate hematologic recovery after Cycle 2 occurred, then the patient began metformin with Cycle 3; if not, the patient was removed from study (Figure 1A). The rationale was to document toxicities from a cycle of non‐metformin‐containing VIT therapy to compare to metformin‐containing cycles for assessment of additive toxicities and possible drug–drug interactions. The trial was amended in 2015 to include metformin at the onset of Cycle 1 and to reduce temozolomide from 100 mg/m^2^/day to 50 mg/m^2^/day orally on Days 1–5 (Figure 1B). Adequate hematologic recovery on Day 21 of Cycle 1 was still required post‐amendment. Metformin dosing was assigned at study entry, beginning with Dose Level 1: 666 mg/m^2^/day divided twice daily on all days of each cycle, equivalent to the typical starting dose of metformin for type II diabetes mellitus. Metformin was purchased commercially through the Moffitt Cancer Center research pharmacy and supplied to sites. Liquid or tablet form of metformin was allowed, and dose rounding was permitted for convenience of administration. Patients were allowed to continue on study for up to 12 cycles. Special dose interruptions were protocol‐mandated to mitigate the risk of metformin‐associated lactic acidosis, including holding metformin for creatinine clearance <60 ml/min/1.73 m^2^, suspected severe hypovolemia, 24 h prior and 48 h post any procedure or anesthesia/sedation requiring the patient to fast, and 24 h prior and 48 h post any procedure or imaging using intravenous contrast.

Temozolomide was required to be given at least one hour prior to the other agents. For Day 1, vincristine was given at least one hour after temozolomide and at least one hour prior to irinotecan. Irinotecan‐induced diarrhea prophylaxis was required with cefpodoxime or cefixime beginning one to two days prior and continuing one to two days post‐irinotecan. Alternative antibiotic prophylaxis was allowed per discretion of the study team. Supportive care for irinotecan early‐ and late‐onset diarrhea was required including use of loperamide, atropine, and octreotide based on symptom timing and intensity.

Tumor resections were allowed on study after the DLT period if deemed clinically indicated by the treating physician. Radiotherapy was not permitted. Subjects were removed from protocol therapy for clinical or radiographic progressive disease, drug‐related adverse events that did not improve or recurred despite dose modifications, refusal of protocol therapy, non‐compliance that in the investigator's opinion precluded ongoing participation, completion of 12 cycles, or if the treating physician determined it was not in the best interest of the patient to continue protocol therapy.

### 2.5 Pharmacokinetic studies

PK samples for metformin were drawn at Hour 0 (pre‐dose) and Hours 6, 12, and 24 following the first metformin dose on Cycle 1 Day 1. Additional PKs were drawn at Hour 0 before the Cycle 1 Day 8 dose and Hour 6 post‐dose. One ml of whole blood was shipped on cold packs overnight to NMS Labs (Willow Grove, PA) where metformin concentration was determined using a previously published assay.^31^ Briefly summarized, pharmacokinetic analysis was conducted using non‐compartmental methods (WinNonlin 8.1; Pharsight). Area under the curve (AUC) for samples obtained up to 12 h were calculated using Linear Trapezoidal Linear Interpolation rule.^32^ The observed time to maximum concentration (Tmax), observed maximum plasma concentration (Cmax) and average plasma concentration at steady‐state (Cssavg) were summarized using descriptive statistics.

### 2.6 Statistical considerations

Patient and clinical characteristics were summarized using descriptive statistics including median and range for continuous measures and proportions and frequencies for categorical measures.

## 3 RESULTS

Twenty‐nine patients consented and were screened (Figure 1C). Three patients failed screening; two due to inadequate renal function and one due to inability to radiographically confirm relapse by the participating institution. Twenty‐six patients were enrolled between October 2012 and June 2019 across seven institutions. The first 11 patients were treated with the initial dosing schema without metformin in Cycle 1 (Figure 1A,C). In this initial cohort, four patients were removed prior to receiving metformin; three due to disease progression, and one due to inability to start metformin by Cycle 3 because of persistent hematologic toxicity. Two additional patients were found to have tumor progression before completing the first metformin‐containing cycle and were not evaluable for DLT. Due to these challenges accruing patients evaluable for DLT and incorporating recommendations of the study's clinical trials oversight committee, the study was amended in 2015 to administer metformin beginning with Cycle 1 and reducing the temozolomide dose of 50 mg/m^2^/day, and 15 more patients were enrolled (Figure 1B,C).

The median age was 13 years (range 2–18 years), and patients received a median of two prior lines of therapy (range 1–5; Table 1). Two patients received prior irinotecan and six patients received prior temozolomide. Overall 109 treatment cycles (with 94 metformin‐containing cycles) were started, and 100 cycles (with 86 metformin‐containing cycles) were completed, with a median of two cycles completed (range 0–12). With dose‐rounding for metformin, the median prescribed dose of metformin was less than 1% different than the protocol‐specified dose (range 0%–15%). No patients underwent tumor resection while on study. The defined dose levels, the number of patients per dose level, and the number evaluable for toxicity and response are in Table 2. Individual patient characteristics in the Table S1.

**TABLE 1:** Demographics and patient characteristics

Characteristics	Number (%)
Total patients	26
Age (years)	
Median	13
Range	2–18
Sex	
Male	12 (46)
Female	14 (54)
Race	
Caucasian	21 (81)
African‐American	4 (15)
Not disclosed	1 (4)
Ethnicity	
Non‐Hispanic	19 (73)
Hispanic or Latino	6 (23)
Not disclosed	1 (4)
Prior lines of therapy	
Median	2
Range	1–5
Patients with prior irinotecan	2
Patients with prior temozolomide	6
Tumor types	
Ewing Sarcoma	8
Rhabdomyosarcoma	3
Atypical teratoid/rhabdoid tumor	3
Osteosarcoma	2
Soft tissue sarcoma	2
Wilms tumor	2
Germ cell tumor	1
Glioblastoma multiforme	1
CNS tumor not otherwise specified	1
CNS medulloepithelioma	1
Anaplastic astrocytoma	1
Brainstem glioma	1

**TABLE 2:** Number of patients receiving treatment by dose level and numbers evaluable for toxicity and response

Dose Level	Metformin Dosing (mg/m^2^/day)	# Patients Treated	# Receiving any Metformin	# Evaluable for DLT	# Patients Experiencing DLT	# Evaluable for Response	# Evaluable for Best Response
1	666	8	5	3	0	5	5
2	999	4	3	3	0	3	2
3	1333	3	3	3	0	3	2
4	1666	3	3	3	0	3	3
5	2000	8	8	6	2	5	4
Totals	—	26	22	18	2	19	16

Abbreviation: DLT, dose‐limiting toxicity.

### 3.1 Toxicity

Of 26 subjects enrolled, 22 received at least one dose of metformin and were included in toxicity analysis (Table 2). All Grade 3 and 4 toxicities from metformin‐ and non‐metformin‐containing cycles, regardless of attribution, are shown in Table 3. Grade 3 and 4 toxicities possibly, probably or definitely attributed to metformin are shown in the Table S2. Grade 3 and 4 hematologic toxicities in all metformin‐containing cycles regardless of attribution included anemia (16%), thrombocytopenia (9.6%), and neutropenia (29.8%). Grade 3 and 4 thrombocytopenia occurred more frequently in non‐metformin‐containing cycles (20%), which may correlate with the higher dose of temozolomide given prior to the trial amendment, while Grade 3 and 4 neutropenia was more common in metformin‐containing cycles. As expected, a significant portion of non‐hematologic adverse events were gastrointestinal, including abdominal pain, diarrhea, dehydration, nausea, vomiting, and weight loss. Notably, Grade 3 and 4 diarrhea occurred more frequently in metformin‐containing cycles (6.4% vs. none in non‐metformin‐containing cycles). No patient died on study from treatment related toxicity. Six patients died in the 30‐day follow‐up period after treatment discontinuation, five from progressive disease and one from pulmonary hemorrhage, which was deemed unrelated to study treatment by the investigator. Seven metformin‐containing treatment cycles were delayed due hematologic toxicity.

**TABLE 3:** Grade 3 and 4 toxicities, regardless of attribution, and percent of cycles in which each toxicity occurred for metformin‐containing and non‐metformin‐containing cycles of therapy

Toxicity group	Toxicity type	Metformin (*n* = 94)			No Metformin (*n* = 15)		
		Grade 3	Grade 4	% Cycles	Grade 3	Grade 4	% Cycles
Blood and lymphatic system disorders	Anemia	15		16.0	1		6.7
	Febrile neutropenia	2		2.1	1		6.7
Ear and labyrinth disorders	Ear pain				1		6.7
	Hearing impaired	1		1.1			
Gastrointestinal disorders	Abdominal pain	2		2.1			
	Diarrhea	5	1	6.4			
	Nausea	2		2.1	1		6.7
	Vomiting	2		2.1			
General disorders and administration site conditions	Fatigue				1		6.7
	Fever				1		6.7
	Gait disturbance	1		1.1			
	Pain	1		1.1			
Immune system disorders	Allergic reaction	1		1.1			
Infections and infestations	Enterocolitis infectious				1		6.7
Investigations	Alanine aminotransferase increased	7		7.4			
	Aspartate aminotransferase increased	2		2.1			
	Lymphocyte count decreased	1	1	2.1			
	Neutrophil count decreased	17	11	29.8		1	6.7
	Platelet count decreased	4	5	9.6	1	2	20.0
	Weight loss	2		2.1			
	White blood cell decreased	9	1	10.6			
Metabolism and nutrition disorders	Anorexia	2		2.1			
	Dehydration	5	1	6.4	1		6.7
	Hypokalemia	3		3.2			
	Hypomagnesemia	2		2.1			
	Hyponatremia				1		6.7
	Hypophosphatemia	1		1.1			
Musculoskeletal and connective tissue disorders	Back pain	1		1.1			
	Generalized muscle weakness	1		1.1			
	Pain in extremity	1		1.1			
Nervous system disorders	Aphonia	1		1.1			
	Depressed level of consciousness	2		2.1			
	Dysarthria	1		1.1			
	Headache	2		2.1			
	Hydrocephalus	1		1.1			
	Nervous system disorders ‐ Other, specify	1		1.1			
	Peripheral motor neuropathy	1		1.1			
	Seizure		1	1.1			
Psychiatric disorders	Confusion	1		1.1			
	Hallucinations	1		1.1			
Renal and urinary disorders	Hematuria				2		13.3
Respiratory, thoracic and mediastinal disorders	Hypoxia	1		1.1	1		6.7
Vascular disorders	Hypotension					1	6.7

Eighteen patients were evaluable for DLT (Table 2). During the DLT period, all DLT‐evaluable patients received all prescribed doses of VIT, and the median metformin dose compliance was 100% (range 95%–100%). No DLTs occurred in Dose Levels 1–4. Two DLTs occurred at Dose Level 5 (metformin 2000 mg/m^2^/day); both were Grade 3 diarrhea requiring hospitalization and more than 48 h of IV hydration. After completion of Dose Level 5, the study's clinical trials oversight committee recommended closure of the trial. One patient was removed from study after DLT, while the other patient completed Cycle 1 and three subsequent cycles after a metformin dose reduction to 1666 mg/m^2^/day. No other patients required metformin dose reductions during the study. The MTD was not definitively determined due to lack of six patients treated at Dose Level 4. Due to the tolerability at this dose level, the tolerability of this dose in a Dose Level 5 patient after dose reduction, we propose Dose Level 4 as the RP2D.

### 3.2 Responses

**FIGURE 2:**
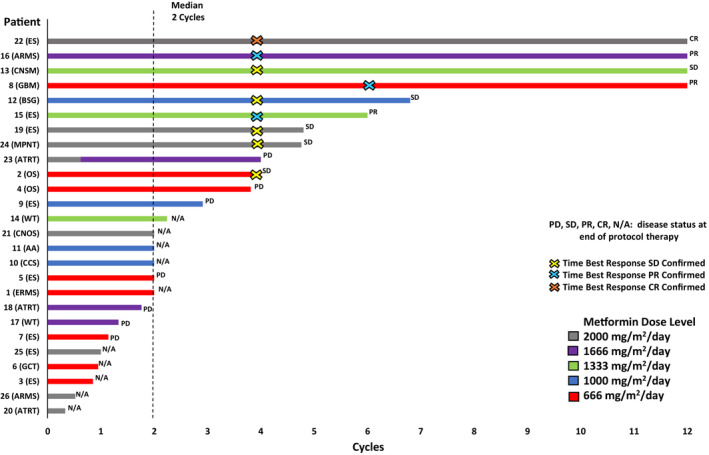
Swimmer's plot of 26 patients treated on study, with timing of overall best response and disease status at end of protocol therapy shown. AA, anaplastic astrocytoma; ARMS, alveolar rhabdomyosarcoma; ATRT, atypical teratoid/rhabdoid tumor of CNS; BSG, brainstem glioma; CCS, clear cell sarcoma of the kidney; CNOS, CNS tumor not otherwise specified; CNSM, central nervous system medulloepithelioma; CR, complete response; ERMS, embryonal rhabdomyosarcoma; ES, Ewing sarcoma; GBM, glioblastoma multiforme; GCT, germ cell tumor; N/A, not applicable as best overall response was unable to be determined; OS, osteosarcoma; PD, progressive disease; PR, partial response; SD, stable disease; WT, Wilms Tumor.

Nineteen patients achieved the threshold metformin exposure defined in the methods section or progressed after initiation of metformin making them evaluable for response, with 16 patients evaluable for best overall response (Figure 1C). Best overall responses included one patient with CR, three patients with PR, five patients with SD, and seven patients with PD. The CR was a 14‐year‐old with Ewing sarcoma who achieved CR after two cycles of chemotherapy, and PRs were seen in patients with glioblastoma multiforme, Ewing sarcoma, and alveolar rhabdomyosarcoma. Four patients received the maximum 12 cycles of therapy. Response timing and length of therapy by patient is shown in Figure 2.The three patients not included in best overall response were a patient with Wilms tumor with PR on first imaging but whose family refused to continue protocol therapy until second imaging, a patient with clear cell sarcoma of the kidney who had SD on first imaging but came off study prior to second imaging per physician discretion to pursue alternative therapy, and a patient with CNS tumor not otherwise specified with SD on first imaging who was removed prior to second imaging per physician discretion for concerns about ongoing metformin compliance.

### 3.3 Pharmacokinetics

Twenty patients had sufficient PK sample collection for metformin PK analysis on Cycle 1 Day 1. As shown in Table 4, steady‐state concentrations varied minimally between cohorts, independent of dose. The AUC_0‐12_ of metformin at the 1666 mg/m^2^ (RP2D cohort) was 1615 h × ng/ml. Average plasma concentration at steady state for evaluable patients at the RP2D was 404 ng/ml. (Table 4).

**TABLE 4:** Pharmacokinetic analysis by dose level

Dose level	N(age range, years)	T_max (hr)_	C_max_ (ng/ml)	AUC_(0–12 h)_ (hr × ng/ml)	C_SS_^avg^ (ng/ml)
666 mg/m^2^/day	5 (5–18)	8 ± 4	763 ± 268	4088 ± 3254	413 ± 158
1000 mg/m^2^/day	3 (6–15)	6 ± 0	440 ± 27	2530 ± 987	297 ± 70
1333 mg/m^2^/day	2 (8–16)	7 ± 1	730 ± 523	2920 ± 537	516 ± 431
1666 mg/m^2^/day	2 (3–6)	6 ± 0	567 ± 134	1615 ± 757	404 ± 157
2000 mg/m^2^/day	7(2–16)	7 ± 2	910 ± 345	3315 ± 1248	557 ± 151

Abbreviations: AUC(0–12 h), area under the curve from 0–12 hr; C_max_, observed maximum plasma concentration; C_ss_^avg^, average plasma concentration at steady‐state; T_max_, observed time to maximum concentration.

## 4 DISCUSSION

This Phase I trial found the MTD was exceeded at Dose Level 5, 2000 mg/m^2^/day of metformin. Both DLTs at Dose Level 5 were diarrhea requiring prolonged hospitalizations with IV fluids. As expected, the majority of toxicities were gastrointestinal, including diarrhea, and hematologic toxicity. In the absence of a conclusive MTD, Dose Level 4 was declared the RP2D.

Recommended metformin dosing for newly‐diagnosed type 2 diabetes mellitus in children 10 years and older is initially 500–1000 mg daily with escalation as high as 1000 mg twice daily.^33^ The MTD/RP2D in our pediatric ALL trial run concurrently with this trial was metformin 1000 mg/m^2^/day alongside a multiagent reinduction backbone.^28^ It is possible that the less intensive VIT backbone with reduced temozolomide dosing allowed higher dose escalation of metformin for this trial. The ALL study also included two events of acidosis reported as DLTs. This study outlined several mitigation measures against the development of metformin‐induced lactic acidosis which may have increased tolerability. Finally, several Grade 3 gastrointestinal toxicities that could be confounded by the VIT backbone and managed with supportive care were excluded from the definition of DLT, which may have contributed to the higher MTD in this study.

This study collected all Grade 3 and 4 toxicities and assessed attribution to metformin, but did not assess attribution to VIT. The toxicities attributed to metformin may have overlapping attribution to VIT or may represent interactions between VIT and metformin. The initial design of this trial adding metformin to VIT in the second cycle was designed to assist in determining additive and overlapping toxicities. For hematologic toxicity, rates of Grade 3 and 4 thrombocytopenia appear higher in the VIT‐only cycles (20% VIT alone vs. 9.6% VIT‐metformin), while Grade 3 and 4 neutropenia was increased in metformin containing cycles (6.7% for VIT alone vs. 29.8%% for VIT‐metformin). Notably, for GI toxicity, rates of Grade 3 and 4 diarrhea appear higher in the VIT‐metformin cycles (0% for VIT alone vs. 6.4% for VIT‐metformin). This increased frequency along with two DLTs of diarrhea requiring prolonged hospitalization with IV fluids indicate diarrhea was an important additional toxicity from VIT‐metformin. Toxicity comparisons between VIT and VIT‐metformin cycles, especially hematologic, must be interpreted with caution, as the dosing of temozolomide was lowered with an amendment after the 11th patient.

An exact toxicity comparison in the literature using the VIT backbone identical to our trial is unavailable, as our study utilized a decrease dose of temozolomide for most patients. Multiple other prior trials and retrospective experiences of VIT have used varying doses, dosing schedules, and administration routes of these agents (e.g. IV vs. oral irinotecan, five‐ vs ten‐day irinotecan dosing schedule, dosing heterogeneity of temozolomide), making direct comparisons to our trial challenging.^4^, ^5^, ^8^, ^9^, ^10^ A Phase I trial of VIT that included a cohort (termed Schedule B) of patients with a five‐day oral dosing strategy of irinotecan with antibiotic prophylaxis reported less hematologic toxicity than that seen on our trial.^8^ Grade 3 and 4 neutropenia occurred in five of 72 Schedule B cycles (6.9%) compared to 28 of 94 metformin‐containing cycles (29.8%) in our trial (Table 3). There was no Grade 3 or 4 anemia reported on Schedule B compared to 15 of 94 cycles (16%) on our trial. While hematologic toxicity is not a frequently‐considered adverse effect with metformin use, there have been reports of metformin lowering neutrophil counts in polycystic ovarian disease and lowering hemoglobin in type 2 diabetes, and its combination with VIT chemotherapy may have exacerbated hematologic toxicity.^34^, ^35^

Comparable to our previous study of metformin in pediatric ALL patients, reported AUC was within 6% at the same dosing cohort (1000 mg/m^2^/day).^28^ The limited sampling scheme affected the PK data from this trial, and 8 out of the 20 PK‐evaluable patients on this study were missing at least one PK time‐point. This limited data may have contributed to the lack of dose‐dependent PK findings on this study. These sampling discrepancies may have occurred because while patients on the ALL study were treated in the hospital, allowing more consistent sample collections, patients on this study were treated outpatient. The sparse data for parameter estimates coupled with varying participants per cohort resulted in non‐correlation between dose and certain PK estimates expected in linear pharmacokinetics, such as AUC and Css. However, previous data supports the linearity between increasing dose levels and these PK estimates for patients who received at least 85% of planned metformin doses.^28^ Additionally, metformin has been described to have a very large volume of distribution, which may translate to a tissue sink, and plasma levels may not necessarily reflect cellular exposure. Tumor concentrations of metformin may have been higher with escalated dosing despite the uninformative PK studies.

Our trial has several limitations. First, because of feasibility difficulties during early enrollment, the dose of temozolomide was lowered from 100 mg/m^2^/day to 50 mg/m^2^/day, decreasing the treatment intensity of VIT. Notably, there were no DLT in the first 11 patients that received full‐dose temozolomide, however we cannot conclude that full‐dose temozolomide is tolerable at the RP2D of metformin from this study. Secondly, with the MTD not conclusively determined, it is possible that our RP2D causes higher levels of toxicity than expected. Finally, while responses were seen in multiple histologies, and five patients experienced confirmed stable disease lasting at least four cycles of therapy, we are unable to determine the activity that metformin adds to VIT in this study, as responses in these histologies has been reported with VIT alone. The Phase I nature of this trial and heterogeneity of tumors enrolled limits the ability to draw conclusions regarding the activity of this regimen for any specific disease.

In conclusion, the addition of metformin to VIT chemotherapy with modified‐dose temozolomide was found to be tolerable, and our RP2D is metformin 1666 m/m^2^/day. Notable toxicities that appeared additive with metformin included diarrhea and neutropenia. Due to the uninformative PKs of this study, future studies of metformin in pediatric tumors would benefit from pharmacodynamic testing of the intratumoral accumulation of metformin. Further assessment of anti‐tumor activity will require evaluation of specific disease cohorts in the context of a Phase II trial.

## AUTHOR CONTRIBUTIONS

**Jonathan Metts:** Data curation (equal); formal analysis (equal); investigation (equal); writing – original draft (lead); writing – review and editing (lead). **Matteo Trucco:** Investigation (equal); writing – original draft (supporting); writing – review and editing (supporting). **Daniel A. A. Weiser:** Investigation (equal); writing – review and editing (supporting). **Patrick A. Thompson:** Investigation (equal); writing – review and editing (supporting). **Eric Sandler:** Investigation (equal); writing – review and editing (supporting). **Tiffany Smith:** Data curation (equal); formal analysis (equal); project administration (equal); resources (equal); writing – review and editing (supporting). **Jessica Crimella:** Data curation (equal); formal analysis (equal); project administration (equal); resources (equal); writing – review and editing (supporting). **Samer Sansil:** Data curation (equal); formal analysis (equal); writing – review and editing (supporting). **Ram Thapa:** Data curation (equal); formal analysis (equal); validation (equal); writing – review and editing (supporting). **Brooke Fridley:** Data curation (equal); formal analysis (equal); methodology (equal); validation (equal); writing – review and editing (supporting). **Nicolas J Llosa:** Investigation (equal); writing – review and editing (supporting). **Thomas Badgett:** Investigation (equal); writing – review and editing (supporting). **Richard G. Gorlick:** Investigation (equal); writing – review and editing (supporting). **Damon R. Reed:** Funding acquisition (equal); investigation (equal); project administration (equal); supervision (equal); writing – review and editing (supporting). **Jonathan B. Gill:** Conceptualization (equal); funding acquisition (equal); investigation (equal); methodology (equal); supervision (equal); writing – original draft (supporting); writing – review and editing (supporting).

## FUNDING INFORMATION

This study was funded by the National Pediatric Cancer Foundation and Rally Foundation.

## CONFLICT OF INTEREST

The authors report no conflicts of interest with this work.

## Supporting information


Tables S1‐S2

## Data Availability

The data that support the findings of this study are available from the corresponding author upon reasonable request.
