# Supplementary material for: A phase I trial of metformin in combination with vincristine, irinotecan, and temozolomide in children with relapsed or refractory solid and central nervous system tumors: A report from the national pediatric cancer foundation
Source: Cancer Med. 2022 Sep 23;12(4):4270–81. doi: 10.1002/cam4.5297 (PMC9972017; doi:10.1002/cam4.5297)
Supplement: Supplementary file 1 — Tables S1‐S2 [file CAM4-12-4270-s001.docx]

**Data Supplement**

**Table S1:** Individual patient information. Patients 1 through 11 were treated with metformin in Cycle 2 or later, while patients 12-26 began metformin in Cycle 1. Abbreviations: DLT: dose-limiting toxicity, ERMS: embryonal rhabdomyosarcoma, ARMS: alveolar rhabdomyosarcoma OS: osteosarcoma, ES: Ewing sarcoma, GCT: germ cell tumor, GBM: glioblastoma multiforme, CCS: clear cell sarcoma of the kidney, CNS: central nervous system, WT: Wilms Tumor, ATRT: atypical teratoid/rhabdoid tumor of CNS, AA: anaplastic astrocytoma, BSG: brainstem glioma, CNSM: CNS medulloepithelioma, CNOS: CNS tumor not otherwise specified, N: no, Y: yes, NE: not evaluable, PD: progressive disease, SD: stable disease, PR: partial response, N/A: not applicable.

|  | **Patient** | **Dose Level (mg/m^2^/day)** | **Metformin Starting Timepoint** | **Diagnosis** | **Age (years) at enrollment** | **Number of Prior Systemic Therapies** | **DLT?** | **# Cycles Completed** | **# Metformin-Containing Cycles Completed** | **Response at 1^st^ Interval Imaging** | **Best**  **Overall Response** |
| --- | --- | --- | --- | --- | --- | --- | --- | --- | --- | --- | --- |
| Metformin 2^nd^ cycle or later | 1 | 666 | Did Not Start | ERMS | 18 | 2 | NE | 2 | 0 | NE | NE |
|  | 2 | 666 | Cycle 2 Day 1 | OS | 16 | 3 | N | 4 | 3 | SD | SD |
|  | 3 | 666 | Did Not Start | ES | 14 | 2 | NE | 0 | 0 | NE | NE |
|  | 4 | 666 | Cycle 3 Day 1 | OS | 18 | 1 | N | 4 | 2 | PD | PD |
|  | 5 | 666 | Cycle 2 Day 1 | ES | 15 | 2 | NE | 2 | 1 | PD | PD |
|  | 6 | 666 | Did Not Start | GCT | 15 | 3 | NE | 1 | 0 | NE | NE |
|  | 7 | 666 | Cycle 2 Day 1 | ES | 5 | 2 | NE | 1 | 0 | PD | PD |
|  | 8 | 666 | Cycle 2 Day 1 | GBM | 18 | 1 | N | 12 | 11 | SD | PR |
|  | 9 | 1000 | Cycle 3 Day 1 | ES | 10 | 1 | N | 3 | 1 | PD | PD |
|  | 10 | 1000 | Cycle 2 Day 1 | CCS | 15 | 4 | N | 2 | 1 | SD | NE |
|  | 11 | 1000 | Did Not Start | AA | 10 | 1 | NE | 2 | 0 | NE | NE |
| Metformin beginning in 1^st^ cycle | 12 | 1000 | Cycle 1 Day 1 | BSG | 6 | 3 | N | 6 | 6 | SD | SD |
|  | 13 | 1333 | Cycle 1 Day 1 | CNSM | 10 | 3 | N | 12 | 12 | SD | SD |
|  | 14 | 1333 | Cycle 1 Day 1 | WT | 8 | 2 | N | 2 | 2 | PR | NE |
|  | 15 | 1333 | Cycle 1 Day 1 | ES | 16 | 1 | N | 6 | 6 | PR | PR |
|  | 16 | 1666 | Cycle 1 Day 1 | ARMS | 9 | 2 | N | 12 | 12 | PR | PR |
|  | 17 | 1666 | Cycle 1 Day 1 | WT | 3 | 1 | N | 1 | 1 | PD | PD |
|  | 18 | 1666 | Cycle 1 Day 1 | ATRT | 6 | 2 | N | 1 | 1 | PD | PD |
|  | 19 | 2000 | Cycle 1 Day 1 | ES | 13 | 1 | N | 4 | 4 | SD | SD |
|  | 20 | 2000 | Cycle 1 Day 1 | ATRT | 2 | 2 | NE | 0 | 0 | NE | NE |
|  | 21 | 2000 | Cycle 1 Day 1 | CNOS | 13 | 4 | NE | 2 | 2 | SD | NE |
|  | 22 | 2000 | Cycle 1 Day 1 | ES | 14 | 1 | N | 12 | 12 | CR | CR |
|  | 23 | 2000 | Cycle 1 Day 1 | ATRT | 5 | 1 | Y | 4 | 4 | SD | PD |
|  | 24 | 2000 | Cycle 1 Day 1 | MPNT | 16 | 3 | N | 4 | 4 | SD | SD |
|  | 25 | 2000 | Cycle 1 Day 1 | ES | 12 | 1 | N | 1 | 1 | NE | NE |
|  | 26 | 2000 | Cycle 1 Day 1 | ARMS | 15 | 5 | Y | 0 | 0 | NE | NE |

**Table S2:** Grade 3 and 4 toxicities deemed possibly, probably, or definitely related to metformin, divided into metformin-containing Cycle 1 (left) and Cycle 2 and beyond (right). The number listed under Grades is the total number of patients experiencing a specific toxicity. The numbers listed under Dose Level signify at what dose level the Grade 3 or 4 toxicities occurred.

| Patients with Adverse Events Attributed to Metformin | | | | | | |
| --- | --- | --- | --- | --- | --- | --- |
|  | **First Metformin-Containing Cycle** | | | **Metformin-Containing Cycle 2+** | | |
| Toxicity Type | **Grade 3** | **Grade 4** | **Dose Level** | **Grade 3** | **Grade 4** | **Dose Level** |
| Abdominal Pain | 1 |  | 5 |  |  |  |
| ALT Increase |  |  |  | 1 |  | 5 |
| Neutrophil Decrease | 1 | 2 | 2,3,3 |  | 1 | 3 |
| Anemia | 1 |  | 2 | 1 |  | 3 |
| Anorexia |  |  |  | 1 |  | 3 |
| Dehydration | 2 |  | 5,5 |  | 1 | 5 |
| Diarrhea | 3 |  | 5,5,5 | 1 | 1 | 3,5 |
| Hypokalemia | 1 |  | 4 | 1 |  | 1 |
| Hypomagnesemia | 1 |  | 4 |  |  |  |
| Nausea | 1 |  | 2 | 1 |  | 5 |
| Platelet Decrease |  |  |  | 1 | 1 | 2,3 |
| Vomiting |  |  |  | 1 |  | 5 |
| WBC Decrease |  |  |  | 1 |  | 3 |
| Weight Loss |  |  |  | 2 |  | 5,5 |
